# Integrative Genomic and Single‐Cell Insights Into Efferocytosis‐Mediated Immune Regulation in Clear Cell Renal Cell Carcinoma

**DOI:** 10.1155/mi/8710699

**Published:** 2025-12-15

**Authors:** Bing Shi, Minghao Deng, Jiakang Ma, Chao Chen, Aijin Peng, Anli Zhu, Rongchao Yang, Zhenhua Jin, Jian Zhu, Mingcong Zhang, Shuijie Shen

**Affiliations:** ^1^ Department of Urology, Nantong Hospital of Traditional Chinese Medicine, Nantong, 226001, China, ntzyy.com; ^2^ Clinical Medical Research Center, Nantong Hospital of Traditional Chinese Medicine, Nantong, 226001, China, ntzyy.com; ^3^ Henan Key Laboratory of Cancer Epigenetics, Cancer Institute, The First Affiliated Hospital, and College of Clinical Medicine of Henan University of Science and Technology, Luoyang, 471003, China, haust.edu.cn; ^4^ Department of Urology, Ningbo Municipal Hospital of Traditional Chinese Medicine (TCM), Affiliated Hospital of Zhejiang Chinese Medical University, Ningbo, Zhejiang, 315010, China, ningbo.gov.cn; ^5^ Department of Urology, Affiliated Lianyungang Clinical College of Nantong University, The Second People’s Hospital of Lianyungang, Lianyungang, China, lygey.com

**Keywords:** clear cell renal cell carcinoma, efferocytosis, machine learning, prognostic model, RAC1, single-cell RNA sequencing

## Abstract

**Background:**

Efferocytosis, the phagocytic clearance of apoptotic cells, plays a key role in tumor progression and immune regulation, but its prognostic significance and molecular mechanisms in clear cell renal cell carcinoma (ccRCC) remain unclear.

**Methods:**

Four efferocytosis‐related pathways were curated, and the pathway activities were quantified in ccRCC. Prognostic genes were identified by univariate Cox regression and used to construct linear survival models with multiple algorithms, with the optimal model selected by cross‐validation. Associations between the risk score and tumor mutational burden (TMB), mutation profiles, and copy number variation (CNV) were subsequently evaluated. Multiomics integration highlighted RAC1 as a key risk gene, which was further examined using single‐cell and spatial transcriptomics (ST) to characterize expression patterns, tumor microenvironment interactions, and pathway enrichments. Protein‐level validation was performed using immunohistochemistry (IHC) data from the Human Protein Atlas.

**Results:**

Efferocytosis pathway activity was upregulated in ccRCC, increased with disease stage, and correlated with poorer survival. The ridge regression‐based prognostic model demonstrated consistent predictive performance across independent datasets and was associated with higher TMB, specific mutation patterns, and increased CNV. Notably, RAC1, identified as the top weighted gene in the model, was overexpressed in association with copy number amplification, showing preferential enrichment in malignant core regions and strong links to oncogenic signaling.

**Conclusion:**

Efferocytosis activation characterizes aggressive ccRCC. The developed prognostic model and identification of RAC1 as a central effector link efferocytosis‐related risk to immune remodeling and oncogenic signaling, providing potential biomarkers and therapeutic targets.

## 1. Introduction

Clear cell renal cell carcinoma (ccRCC) is the most common subtype of renal cancer, characterized by significant clinical and molecular heterogeneity [1]. Despite advances in diagnostic and treatment strategies, still carries a poor prognosis, particularly in advanced stages [2, 3]. This is largely attributed to its inherent biological aggressiveness, therapeutic resistance, and the complexity of the tumor microenvironment [4, 5]. Hence, identifying novel biomarkers and therapeutic targets that reflect underlying biological mechanisms is critical to improving outcomes for ccRCC patients.

Efferocytosis, the process by which apoptotic cells are phagocytosed and cleared by neighboring cells, plays a vital role in maintaining tissue homeostasis and immune regulation [6, 7]. Under physiological conditions, efferocytosis prevents inflammatory responses and maintains immune tolerance by efficiently clearing apoptotic cells [8, 9]. However, in cancer, aberrant activation or dysregulation of efferocytosis contributes to tumor progression through immune evasion, promotion of an immunosuppressive microenvironment, and facilitation of metastatic dissemination [10, 11]. Tumor‐associated macrophages and other stromal cells engaging in efferocytosis have been shown to remodel the tumor immune landscape, influencing cancer cell survival, proliferation, and invasion [12]. Despite these advances, the prognostic implications and molecular mechanisms of efferocytosis in ccRCC remain insufficiently characterized. Exploring efferocytosis‐associated pathways may thus unveil new insights into ccRCC pathogenesis and therapeutic vulnerabilities.

Advances in bioinformatics and high‐throughput sequencing technologies, including bulk RNA sequencing, single‐cell RNA sequencing, and spatial transcriptomics (ST), have provided unprecedented opportunities toinvestigate the complexities of tumor biology at multiple dimensions [13, 14]. Integrated genomic analyses enable comprehensive characterization of molecular alterations, cell‐type‐specific expression patterns, and intercellular communications within the tumor microenvironment, thus facilitating the identification of critical regulatory mechanisms and therapeutic targets.

In this study, we systematically characterize efferocytosis‐related signatures in ccRCC through integrated multiomics analyses, revealing its prognostic relevance and functional importance. Our findings highlight RAC1 as a pivotal gene linking efferocytosis activity with tumor progression, immune remodeling, and oncogenic signaling pathways. These findings provide new insights into ccRCC biology and suggest promising avenues for the development of targeted therapeutic strategies.

## 2. Methods

### 2.1. Data Acquisition and Preprocessing

Bulk RNA‐seq profiles and corresponding clinicopathologic and survival data for ccRCC were retrieved from The Cancer Genome Atlas (TCGA) [15]. Primary tumor and matched/unmatched normal kidney tissues with complete stage and outcome information were retained. Expression values were converted to TPM where applicable and log2(*x* + 1) transformed; gene symbols were harmonized to current HGNC nomenclature. Samples with missing key covariates or excessive zero counts were excluded.

### 2.2. Efferocytosis Gene Sets and Pathway Activity Scoring

Four Gene Ontology (GO) pathways related to efferocytosis were curated from the Molecular Signatures Database (MSigDB version 2025.1) resource, including Efferocytosis (GO:0043277), Regulation of efferocytosis (GO:2000425), Positive regulation of efferocytosis (GO:2000427), and Negative regulation of efferocytosis (GO:2000426). After merging and removing duplicates, a total of 192 unique genes were retained for downstream analyses. The full deduplicated gene list is provided in Supporting Information 1: Table S1 Single‐sample gene set enrichment analysis (ssGSEA) implemented in the GSVA package was used to calculate pathway scores per sample for the four efferocytosis gene sets. For visualization and between‐sample comparison, scores were Z‐standardized within cohort and displayed as heatmaps. Patients were additionally grouped by pathologic stage (I–II vs III–IV) for comparative analyses.

### 2.3. Univariate Cox Analysis

We first performed univariate Cox proportional hazards regression for all 192 genes derived from the four efferocytosis‐related pathways in the TCGA‐KIRC cohort to identify survival‐associated candidates. Genes with *p*  < 0.05 were considered statistically significant for overall survival (OS) and retained for subsequent modeling. Given that the number of efferocytosis‐related genes was limited and the univariate analysis mainly served as an initial screening step rather than the core prognostic evaluation, FDR adjustment was not applied.

### 2.4. Model Construction and Algorithm Evaluation

All prognostically significant genes were directly incorporated as model features without additional dimensionality reduction or preselection to maintain transparency and interpretability. Linear survival models were constructed using multiple algorithms, including Lasso regression, Elastic Net regression (*α* = 0.1–0.9), ridge regression (*α* = 0), stepwise Cox regression (both, forward, backward directions), and CoxBoost. Lasso, Elastic Net, and ridge models were implemented via the glmnet package, with tenfold cross‐validation to determine optimal penalty parameters (λ). Stepwise Cox regression was performed using coxph and stepAIC functions, while CoxBoost modeling employed optimCoxBoostPenalty and cv.CoxBoost to optimize penalty and iteration number. Model coefficients and corresponding gene contributions were extracted from the fitted objects. Predictive performance was assessed by time‐dependent area under the ROC curve (AUC) across multiple datasets and endpoints, and the optimal‐performing algorithm was selected for downstream prognostic evaluation and validation.

### 2.5. Meta‐Analysis and Prognostic Validation

To evaluate the robustness of the prognostic model, internal resampling was performed using bootstrap analysis with 1000 iterations. For each resample, the ridge regression model was refitted and the concordance index (C‐index) was recalculated to assess model performance stability. The risk score from the optimal algorithm was evaluated in multiple independent datasets using univariate Cox regression. Hazard ratios (HRs) were pooled via a random‐effects meta‐analysis model, with heterogeneity assessed by *I* ^2^ statistics. The best cutoff value for stratifying patients into high‐ and low‐risk groups was determined using the “surv_cutpoint” function in the TCGA‐KIRC cohort and subsequently fixed for all external validation datasets to ensure consistency and reduce overfitting.

### 2.6. Tumor Mutational Burden (TMB) and Mutation Count Correlation

Somatic mutation data (MAF format) for ccRCC were obtained from the Genomic Data Commons. TMB was calculated using the “tmb” function in the maftools R package with its built‐in default parameters, and the resulting values were log_10_‐transformed for visualization. Pan‐cancer TMB distributions were plotted, and within KIRC, TMB was compared between high‐ and low‐risk groups (median risk score cut‐off) using the Wilcoxon rank‐sum test. In addition, total, nonsynonymous, and synonymous mutation counts were computed for each sample, and their correlations with the continuous risk score were evaluated using Pearson correlation analysis.

### 2.7. Differentially Mutated Genes and Interaction Analysis

Mutation profiles were summarized using the maftools R package. Differences in gene mutation frequencies between the high‐ and low‐risk groups were evaluated using Fisher’s exact test, and the results were expressed as log odds ratios. All statistical procedures followed the default settings of maftools, which apply Fisher’s exact tests without additional multiple‐testing adjustment. Co‐occurrence and mutual exclusivity among recurrently mutated genes were further examined using the interaction analysis module implemented in maftools. Significance thresholds (*p*  < 0.05 and *p*  < 0.01) were applied according to the package’s default configuration for visualization.

### 2.8. Copy Number Variation (CNV) Analysis

CNV gain and loss frequencies were calculated for each chromosomal region and visualized as percentage plots and GISTIC scores. Patients were stratified into high‐ and low‐risk groups according to the median efferocytosis‐related risk score. The focal and broad CNV burdens were quantified as focal gain load, focal loss load, broad gain load, and broad loss load, which were compared between groups using the Wilcoxon rank‐sum test.

### 2.9. Single‐Cell Transcriptomic Analysis of RAC1

Single‐cell RNA sequencing data for ccRCC (GSE159115) were obtained from the Tumor Immune Single‐Cell Hub 2 (TISCH2) database and data preprocessing followed TISCH2’s standardized workflow [16, 17]. Gene expression was normalized, highly variable genes were identified, and principal component analysis (PCA) followed by Uuniform manifold approximation and projection (UMAP) was performed for dimensionality reduction and clustering. Cell types were annotated into major lineages,including immune, malignant, and stromal compartments,based on canonical markers provided by TISCH2. Cell–cell communication analysis was conducted with CellChat, stratifying malignant cells into RAC1^+^ and RAC1^-^ subpopulations according to the median RAC1 expression. Communication counts (number of significant ligand–receptor interactions) and weights (aggregate interaction strength) were computed for each senderreceiver pair, and outgoing/incoming signaling patterns as well as pathway‐level activities were summarized by heatmaps and dot plots with FDR annotations.

### 2.10. ST Analysis of RAC1 Expression and Microenvironmental Interactions

ST data for ccRCC were obtained from the Sparkle database (https://grswsci.top/), including all available sections from GSE175540. RAC1 expression was first evaluated across all samples, and four FFPE sections (GSM5924033, GSM5924035, GSM5924036, GSM5924040) with well‐preserved histological architecture were selected for detailed analysis. Cell types were annotated via Seurat label transfer using a ccRCC scRNA‐seq reference from the same database, and RAC1 spatial expression was mapped relative to annotated cell types [18]. Tumor core and peripheral regions were delineated histologically, with RAC1 expression compared by Wilcoxon rank‐sum test. Cell–cell communication networks were inferred using CellChat, and correlations between RAC1 expression and cell type–specific interaction metrics were visualized by heatmaps and chord diagrams.

### 2.11. Multidimensional Characterization of RAC1‐Linked Immune and Oncogenic Features

A curated immune checkpoint gene set, including costimulatory and coinhibitory molecules, ligands, receptors, cell adhesion molecules, and antigen presentation genes, was analyzed for its correlation with RAC1 expression. Oncogenic pathway activities were quantified by ssGSEA using hallmark and curated gene sets encompassing angiogenesis, apoptosis, cell cycle regulation, DNA damage and repair, epithelial‐to‐mesenchymal transition (EMT), inflammation, metastasis, proliferation, oxidative stress, and stemness [19].

### 2.12. Protein Expression Validation

Immunohistochemistry (IHC) images for RAC1 in ccRCC were retrieved from the Human Protein Atlas. Cases were categorized by staining intensity (low or medium) according to database annotations.

## 3. Results

### 3.1. Efferocytosis Activity Is Elevated in ccRCC and Predicts Poorer Survival

In TCGA‐ccRCC cohort, ssGSEA‐derived efferocytosis activity displayed a consistent elevation pattern across malignant contexts. Heatmaps analyses revealed globally higher scores in tumors samples compared with normal samples, with a further increase from early (Stage I–II) to advanced disease (Stage III–IV), highlighting progressive activation during tumor evolution (Figure 1A). Consistent with these observations, tumors exhibit significantly higher pathway scores than normal samples (Figure 1B). Logistic regression analysis based on individual pathway scores show close agreement between apparent and bootstrap bias‐corrected calibration curves with high concordance, indicating high concordance and reliable discrimination between tumor and normal samples (Figure 1C). Stage‐stratified comparisons confirm higher pathway activity in stage III–IV than in stage I‐II (Figure 1D). Moreover, Kaplan–Meier analyses revealed that patients with elevated pathway activity (using the optimal cutoff) had significantly worse OS, showing elevated HRs and clear survival separation by log‐rank test across key efferocytosis‐related pathways (Figure 1E). Collectively, these data indicate that heightened efferocytosis pathway activity is a robust hallmark of ccRCC that scales with disease stage, distinguishes tumors from normal tissue, and portends inferior survival.

Figure 1Efferocytosis‐related pathway activity in ccRCC. (A) Heatmaps of Z‐standardized ssGSEA scores for four efferocytosis‐related GO pathways (Efferocytosis, Regulation of efferocytosis, Positive regulation of efferocytosis, Negative regulation of efferocytosis) in normal versus tumor tissues (top) and in Stage I–II versus Stage III–IV tumors (bottom). (B) Violin/box plots comparing pathway scores between normal and tumor samples. (C) Calibration curves for logistic regression models using each pathway score to classify tumor versus normal status, showing apparent and bootstrap bias‐corrected fits with corresponding concordance indices (C‐index). (D) Violin/box plots comparing pathway scores between early (Stage I–II) and advanced (Stage III–IV) stage. (E) KaplanMeier curves for overall survival stratified by high versus low efferocytosis pathway activity.

**Figure:**
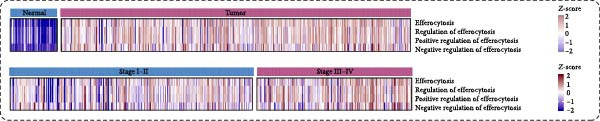
(A)

**Figure:**
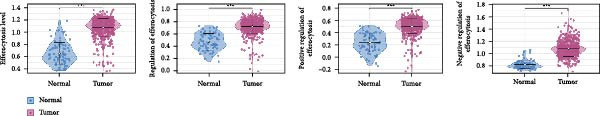
(B)

**Figure:**
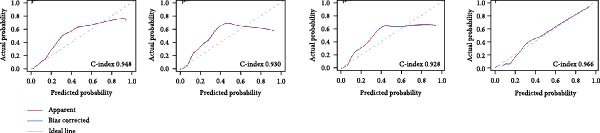
(C)

**Figure:**
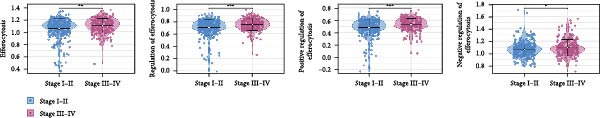
(D)

**Figure:**
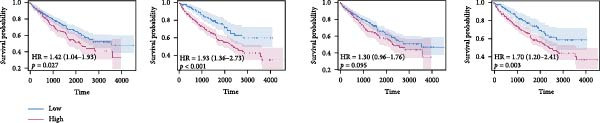
(E)

### 3.2. Development and Validation of an Efferocytosis‐Related Linear Prognostic Model

Univariate Cox analysis identified 26 genes significantly associated with OS in ccRCC, including adverse prognostic genes such as RAC1, AXL, and XKR8, as well as protective genes such as ITGAV and RHOBTB1 (Figure 2A). These genes were used directly as model features to construct linear survival models with multiple algorithms. Among the approaches tested, ridge regression consistently achieved the highest and most stable time‐dependent AUCs across TCGA‐KIRC and independent validation datasets, outperforming Lasso, Elastic Net, stepwise Cox, and CoxBoost (Figure 2B). To facilitate reproducibility, the complete set of gene coefficients used to compute the efferocytosis‐related risk score has been provided in Supporting Information 1: Table S2. The coefficient heatmap revealed that risk genes carried positive weights, while protective genes carried negative weights, with several genes demonstrating stable contributions across algorithms (Figure 2C). Using the ridge‐derived risk score, univariate Cox analyses across multiple datasets showed that a higher score was consistently associated with poorer OS. Meta‐analysis demonstrated an overall significant association (pooled HR = 3.867, 95% CI 1.729–8.648; *p*  < 0.01), but considerable heterogeneity (*I* ^2^ = 98%) indicated that effect sizes varied across datasets, likely reflecting differences in sequencing platforms and patient compositions (Figure 2D). Kaplan–Meier curves further demonstrated significantly shorter OS in the high‐risk group compared with the low‐risk group in the TCGA‐KIRC, E‐MTAB‐1980, and GSE22541 cohorts (all *p*  < 0.05), supporting the prognostic accuracy and generalizability of the efferocytosis‐related model (Figure 2E).

Figure 2Development and validation of an efferocytosis‐related linear prognostic model in ccRCC. (A) Forest plot of univariate Cox proportional hazards regression for 192 efferocytosis‐related genes in the TCGA‐KIRC cohort, identifying 26 genes significantly associated with overall survival (*p*  < 0.05). (B) Heatmap of time‐dependent area under the ROC curve (AUC) values for multiple linear survival modeling algorithms—Lasso, Elastic Net, Ridge, stepwise Cox, and CoxBoost—across the TCGA‐KIRC and independent validation datasets. (C) Heatmap of regression coefficients for each gene across modeling algorithms (top) and bar plot of coefficients from the optimal Ridge regression model (right), with positive weights indicating risk genes and negative weights indicating protective genes. (D) Forest plot summarizing univariate Cox regression of the Ridge‐derived risk score in multiple datasets, with pooled hazard ratio (HR) estimated by a random‐effects meta‐analysis; heterogeneity quantified by *I*² statistic. (E) KaplanMeier survival curves for overall survival in the TCGA‐KIRC, E‐MTAB‐1980, and GSE22541 cohorts stratified by high versus low Ridge‐derived risk score (best cut‐off), with shaded 95% confidence intervals, Cox model HRs, and log‐rank *p* values annotated.

**Figure:**
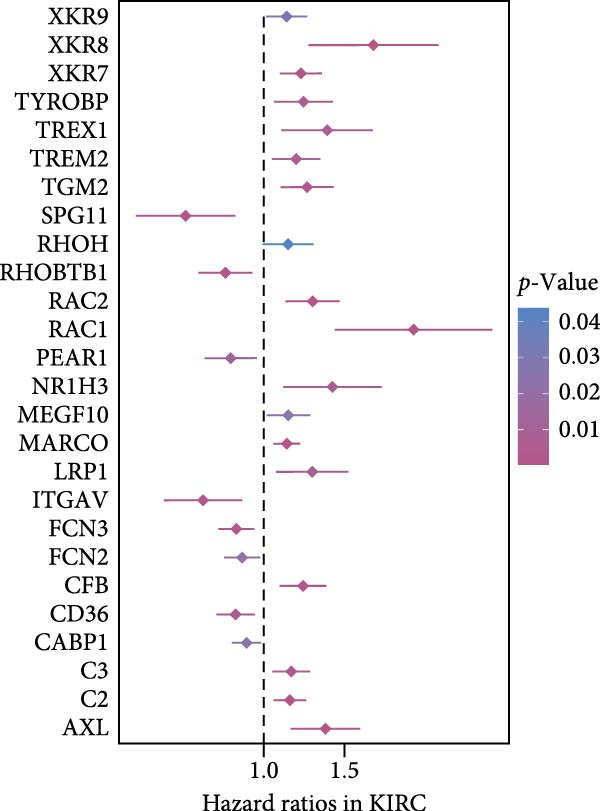
(A)

**Figure:**
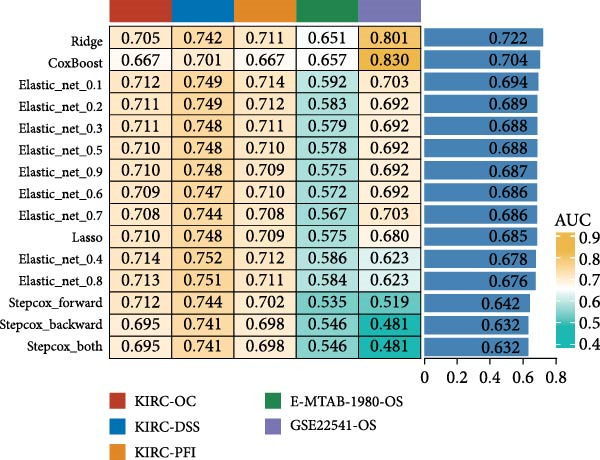
(B)

**Figure:**
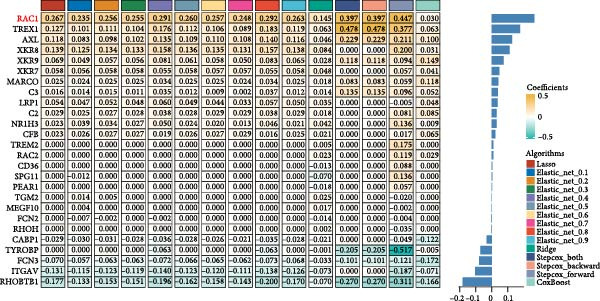
(C)

**Figure:**
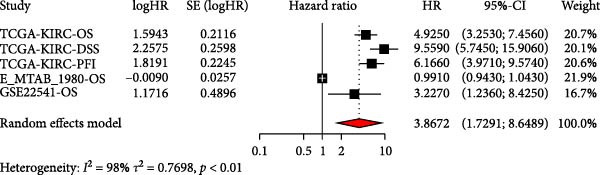
(D)

**Figure:**
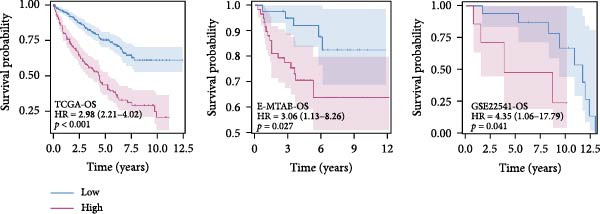
(E)

### 3.3. Relationship of the Efferocytosis‐Related Signature With Mutation Landscape in ccRCC

Pan‐cancer analysis revealed heterogeneous TMB distributions across 33 TCGA cancer types, with ccRCC showing a relatively modest median TMB compared to other malignancies (Figure 3A). Within the ccRCC cohort, patients classified as high‐risk according to the efferocytosis‐related prognostic model displayed significantly higher TMB than those in the low‐risk group (*p*  = 0.039) (Figure 3B). Continuous risk scores showed a modest but positive correlation with total mutation counts (*R* = 0.13, *p*  = 0.017), nonsynonymous mutation counts (*R* = 0.14, *p*  = 0.011), and synonymous mutation counts (*R* = 0.11, *p*  = 0.039) (Figure 3C‐E). Differential mutation analysis further identified several genes with significantly different mutation frequencies between high‐ and low‐risk groups, including higher mutation prevalence of BAP1, MTOR, and TRMT1L in the high‐risk group (Figure 3F). Co‐occurrence analysis revealed significant positive associations among multiple frequently mutated genes, with no evidence of mutual exclusivity (Figure 3G). Collectively, these findings indicate that elevated efferocytosis‐related risk scores are associated with increased mutational burden and distinct mutation patterns in ccRCC.

Figure 3Association of the efferocytosis‐related risk score with tumor mutational burden and mutation landscape in ccRCC. (A) Pan‐cancer distribution of tumor mutational burden (TMB) across 33 TCGA cancer types. (B) Comparison of TMB between high‐ and low‐risk groups in ccRCC, defined by the median efferocytosis‐related risk score. (C–E) Pearson correlation analyses between the continuous risk score and total mutation counts (C), nonsynonymous mutation counts (D), and synonymous mutation counts (E), with correlation coefficients (R) and P values indicated. (F) Differentially mutated genes between high‐ and low‐risk groups identified by Fisher’s exact test, with log odds ratios indicating the direction and magnitude of association. (G) Co‐occurrence and mutual exclusivity analysis of frequently mutated genes; blue shading indicates co‐occurrence, and statistical significance is annotated.

**Figure:**
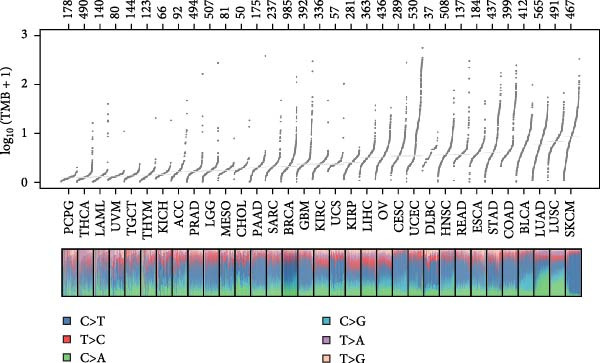
(A)

**Figure:**
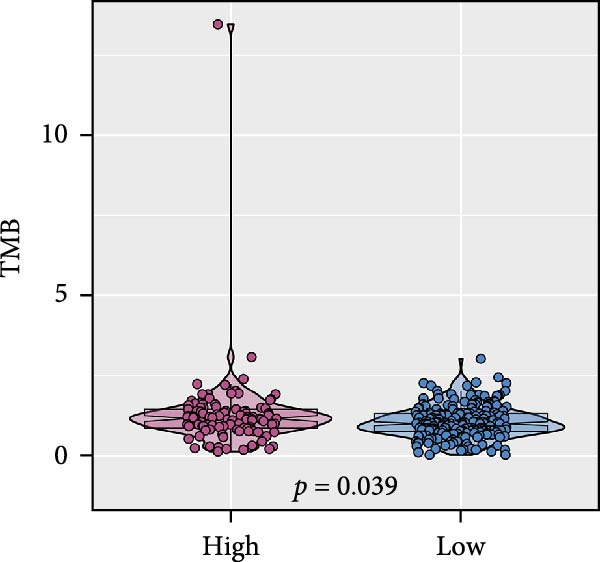
(B)

**Figure:**
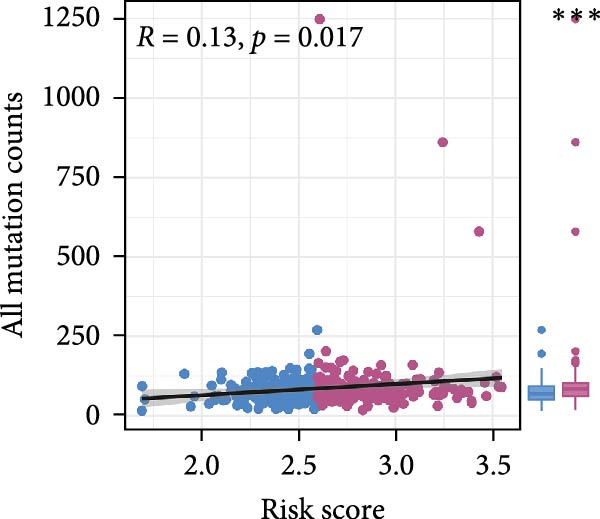
(C)

**Figure:**
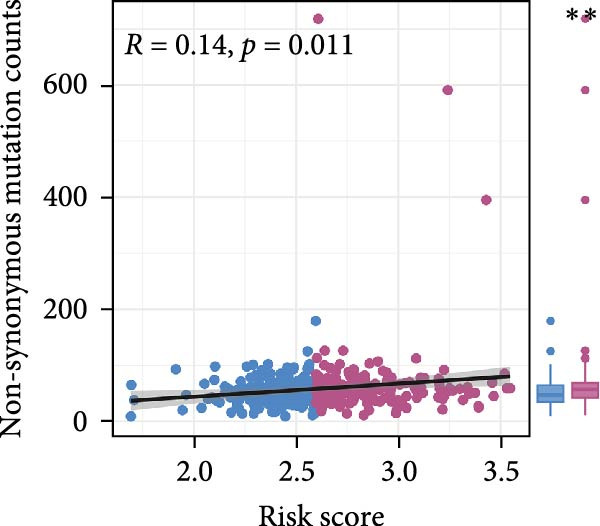
(D)

**Figure:**
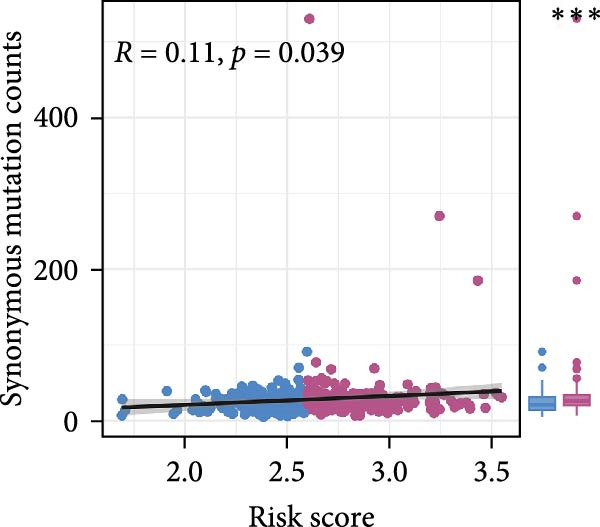
(E)

**Figure:**
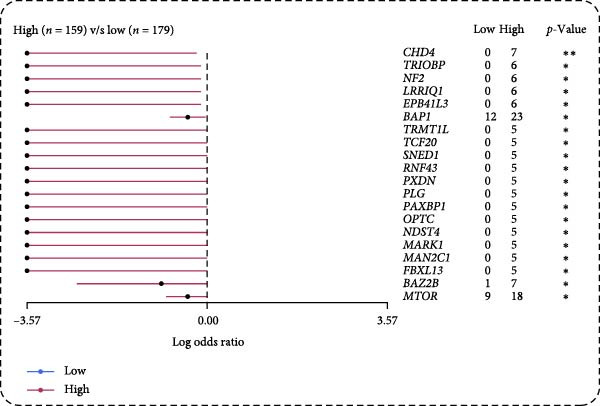
(F)

**Figure:**
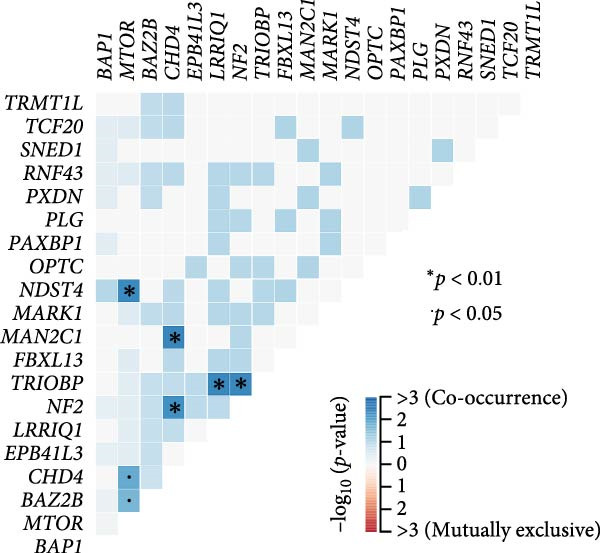
(G)

### 3.4. RAC1‐Centered CNV Alterations Reflect Elevated Genomic Instability in High‐Risk Samples

Genome‐wide CNV profiling revealed characteristic copy number gains and losses in ccRCC, including broad losses on Chromosomes 3 and 14 and broad gains on cChromosomes 5, 7, and 8 (Figure 4A, B). When stratified by efferocytosis‐related risk score, the high‐risk group exhibited significantly greater CNV burden across all categories, including focal gain load (*p*  = 1.1 × 10^−8^), focal loss load (*p*  = 7.8 × 10^−12^), broad gain load (*p*  = 1.0 × 10^−13^), and broad loss load (*p*  = 1.8 × 10^−15^), compared with the low‐risk group (Figure 4C). Notably, for RAC1, the top‐weighted gene in our prognostic model, high‐risk patients showed markedly higher fractions of genome altered, gained, and lost at the RAC1 locus (Figure 4D). Furthermore, RAC1 copy number (GISTIC score) was positively correlated with its mRNA expression (Spearman *r* = 0.40, *p*  = 2.09 × 10^−21^), suggesting that CNV‐driven amplification may contribute to transcriptional upregulation of this key efferocytosis‐related risk gene (Figure 4E).

Figure 4Association of efferocytosis‐related risk score with CNV burden and RAC1 genomic alterations. (A) Percentage plot of copy number gains and losses across all chromosomes in TCGA‐KIRC. (B) GISTIC 2.0 score plot showing the significance of recurrent CNV events. (C) Comparison of focal gain load, focal loss load, broad gain load, and broad loss load between high‐ and low‐risk groups defined by the efferocytosis‐related prognostic model. (D) Fraction of genome altered (FGA) and fraction of genome gained or lost (FGG/FGL) for the RAC1 locus in high‐ versus low‐risk groups, highlighting the elevated CNV burden in the high‐risk group. (E) Spearman correlation between RAC1 GISTIC copy number values and RSEM‐normalized mRNA expression.

**Figure:**
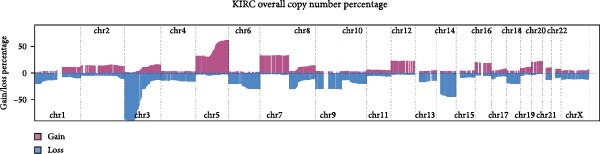
(A)

**Figure:**
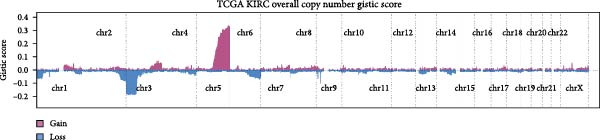
(B)

**Figure:**
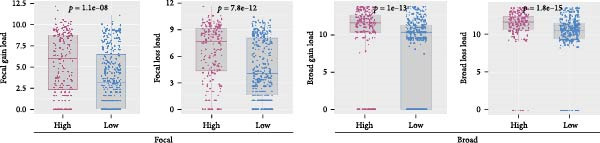
(C)

**Figure:**
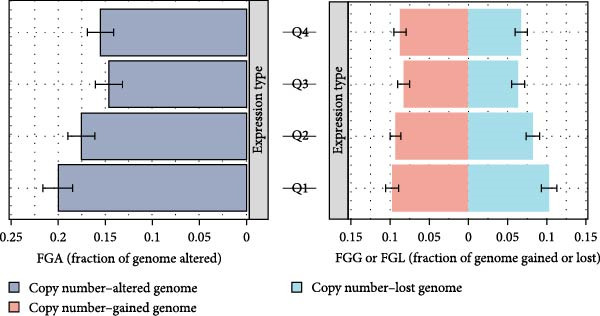
(D)

**Figure:**
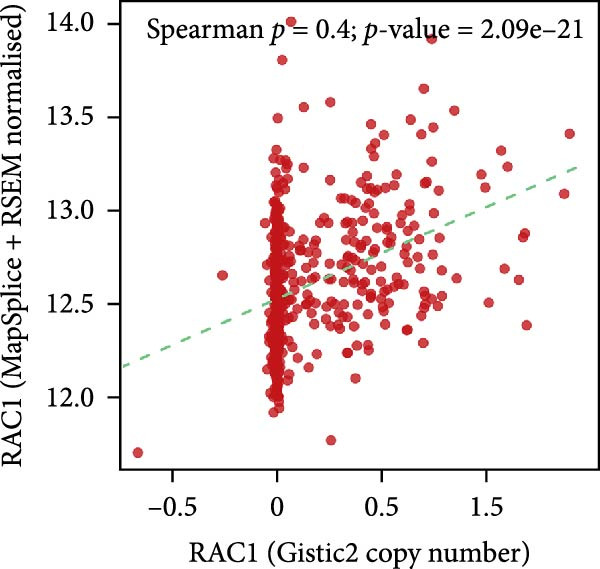
(E)

### 3.5. RAC1 Is Enriched in malignant Cells and Reshapes Intercellular Communication

Unsupervised UMAP revealed distinct cellular clusters across GSE159115 (Figure 5A), which were annotated into major lineages including CD8^+^ T cells, endothelial and epithelial cells, erythroblasts, malignant cells, monocytes/macrophages, pericytes, and plasma cells (Figure 5B). RAC1 exhibited heterogeneous but discernible enrichment within malignant and select stromal/immune populations, as shown by expression and density projections (Figure 5C, D). Quantitatively, RAC1 levels differed significantly among cell types (overall *p*  < 0.001), with higher expression in malignant and endothelial compartments (Figure 5E, F). CellChat analysis demonstrated that RAC1 expression status stratifies malignant‐cell communication: RAC1^+^ malignant cells engaged in a greater number of significant interactions (counts) with multiple partners than RAC1^-^ counterparts, and also exhibited higher aggregate interaction strength (weight) across networks (Figure 5G). Correspondingly, outgoing and incoming signaling patterns highlighted intensified pro‐tumorigenic pathways originating from RAC1^+^ malignant cells (Figure 5H). At the pathway level, malignant cells showed enrichment of proliferative, EMT, and survival‐related signaling, whereas immune and stromal compartments displayed distinct functional pathway profiles (Figure 5I). These findings indicate that RAC1^+^ malignant cells form denser communication networks with immune and stromal populations, suggesting enhanced microenvironmental signaling activity.

Figure 5Single‐cell profiling of RAC1 expression and cell‐cell communication. (A) UMAP of single cells showing unsupervised clustering. (B) UMAP annotated by major lineages, including CD8⁺ T cells, endothelial cells, epithelial cells, erythroblasts, malignant cells, monocytes/macrophages, pericytes, and plasma cells. (C) UMAP colored by RAC1 expression level. (D) Kernel density map illustrating the spatial distribution of RAC1 expression. (E) Violin/box plots comparing RAC1 expression among annotated cell types. (F) RAC1 expression across immune, malignant, and stromal compartments. (G) CellChat analysis comparing RAC1⁺ versus RAC1⁻ malignant cells: left, number of significant ligand‐receptor interactions (counts); right, overall communication strength (weight). (H) Heatmaps summarizing outgoing and incoming signaling patterns across annotated cell types. (I) Dot plot summarizing pathway‐level communication activity across cell types, with dot size representing statistical significance and color indicating relative activity.

**Figure:**
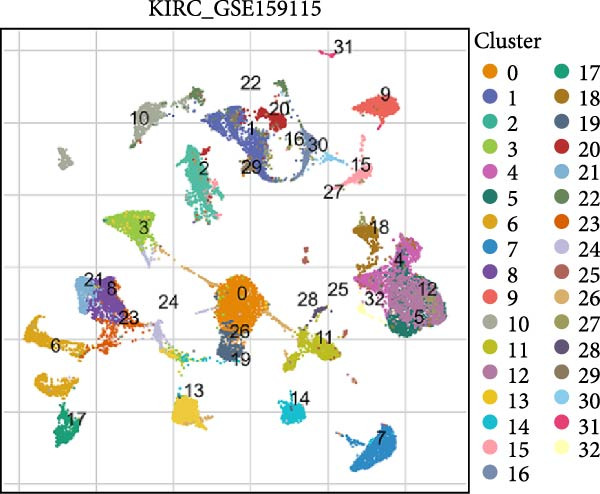
(A)

**Figure:**
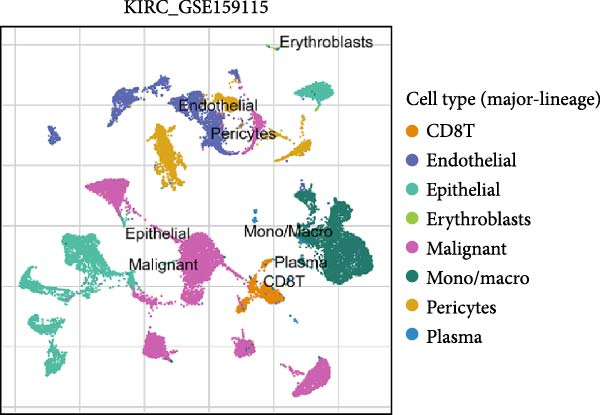
(B)

**Figure:**
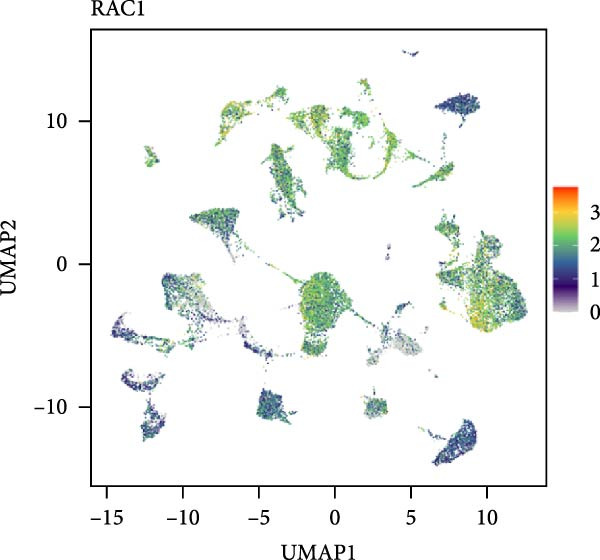
(C)

**Figure:**
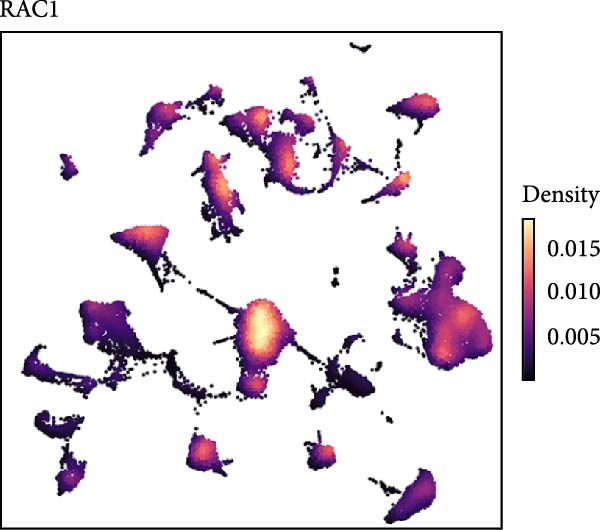
(D)

**Figure:**
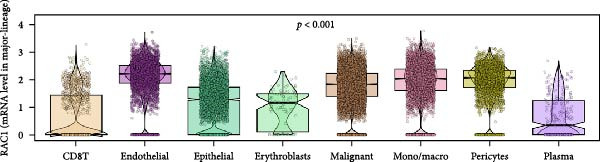
(E)

**Figure:**
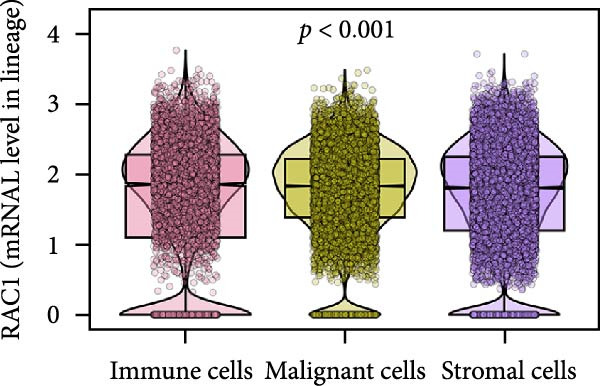
(F)

**Figure:**
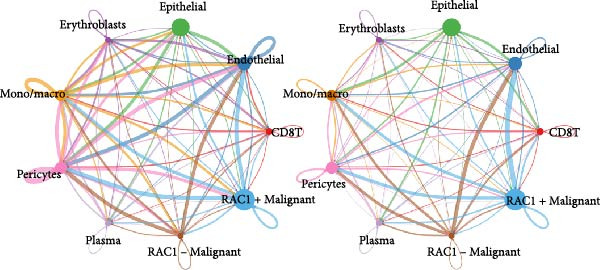
(G)

**Figure:**
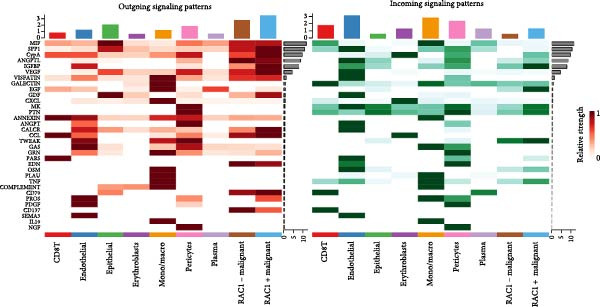
(H)

**Figure:**
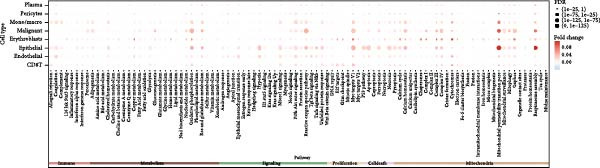
(I)

### 3.6. Spatial Distribution of RAC1 and Its Association With the Tumor Microenvironment

Across the GSE175540 cohort, RAC1 expression exhibited marked variability among samples, with several tumor‐rich sections showing elevated transcript abundance (Figure 6A). In the four sections with well‐preserved histological architecture (GSM5924033, GSM5924035, GSM5924036, and GSM5924040), cell type annotation revealed distinct compartmentalization of malignant, immune, and stromal populations (Figure 6B). RAC1 spatial mapping demonstrated preferential localization of high expression zones within malignant cell‐enriched regions (Figure 6C). Tumor segmentation analysis confirmed significantly higher RAC1 expression in tumor core regions compared with peripheral areas across all four sections (all *p*  < 0.001) (Figure 6D, E). Correlation‐based cell–cell communication analysis indicated that RAC1 expression was positively associated with interaction intensities involving malignant cells, CD8^+^ T cells, and macrophages, suggesting that RAC1‐high niches may foster more active intercellular signaling (Figure 6F). These results highlight a spatially restricted, tumor‐centric pattern of RAC1 expression in ccRCC and its potential link to microenvironmental communication dynamics.

Figure 6Spatial transcriptomics reveals core‐localized RAC1 expression and its association with microenvironmental dynamics. (A) Heatmap showing *Z*‐score‐normalized RAC1 expression across all samples in GSE175540. (B) Spatial transcriptomics (ST) maps of four FFPE sections (GSM5924033, GSM5924035, GSM5924036, GSM5924040) with cell type annotation. (C) Spatial distribution of RAC1 expression overlaid on the same sections; red indicates higher expression. (D) Histology‐guided segmentation of tumor core and peripheral regions. (E) Quantitative comparison of RAC1 expression between tumor core and peripheral regions in each section. (F) Correlation‐based cell‐cell communication analysis showing associations between RAC1 expression and interaction strength for each annotated cell type.

**Figure:**
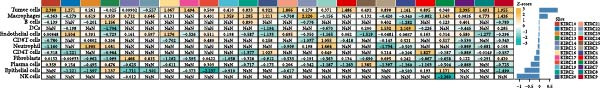
(A)

**Figure:**
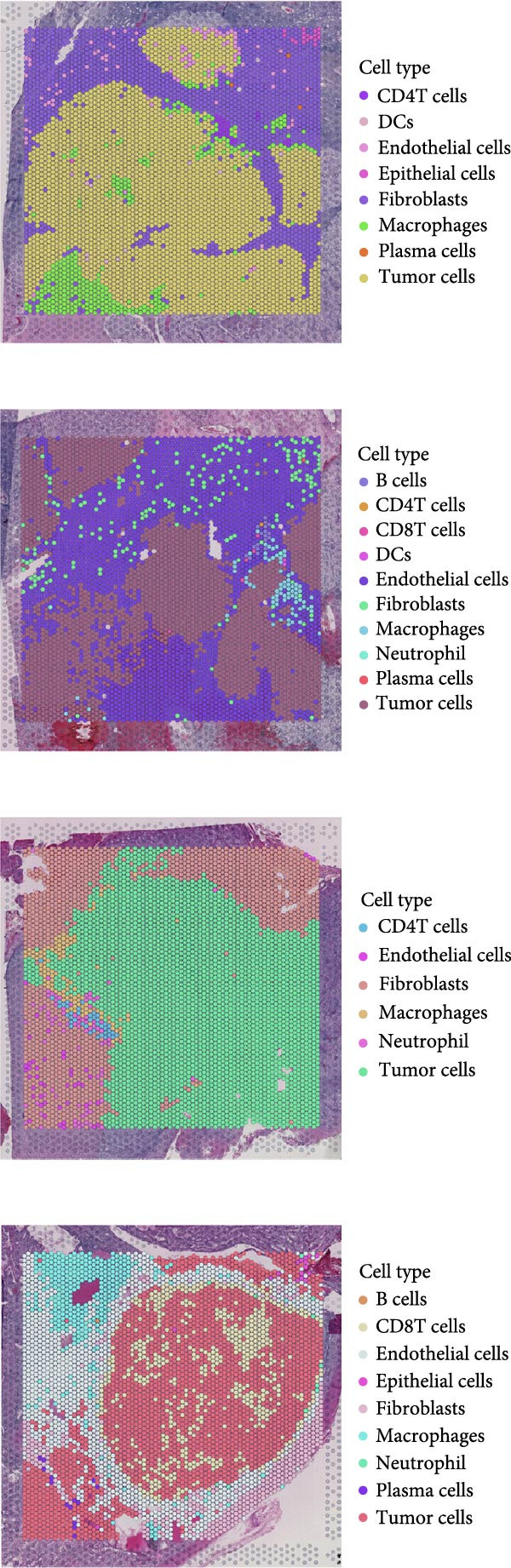
(B)

**Figure:**
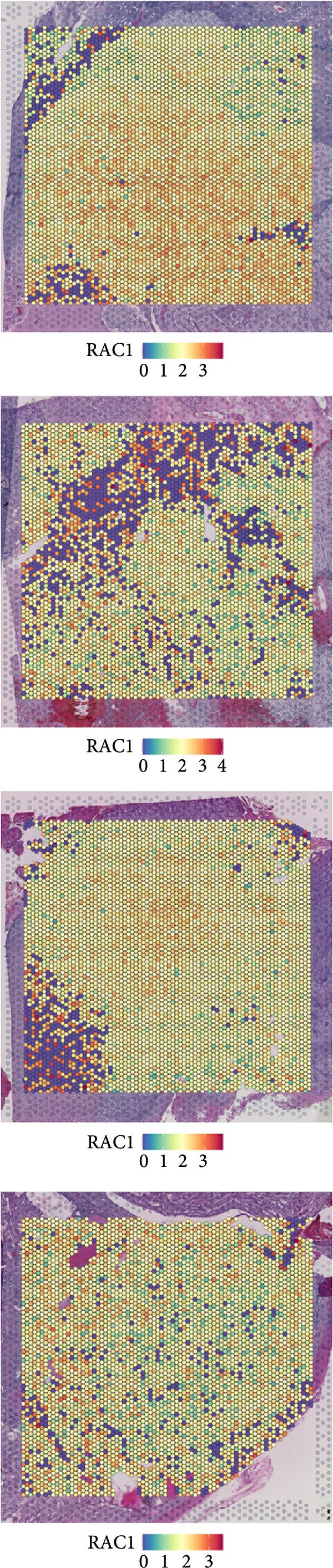
(C)

**Figure:**
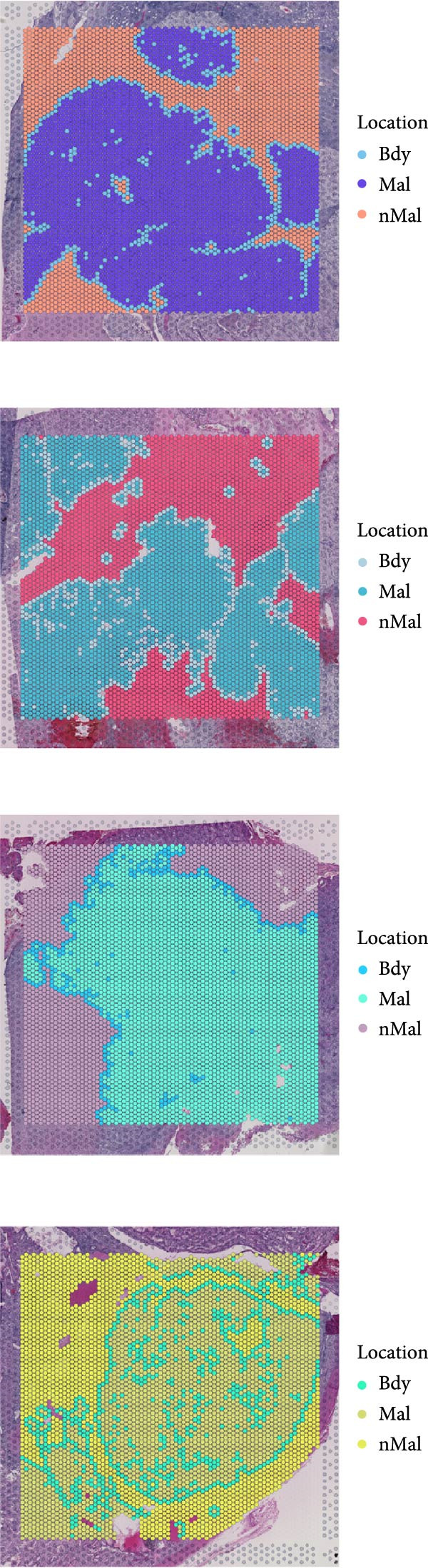
(D)

**Figure:**
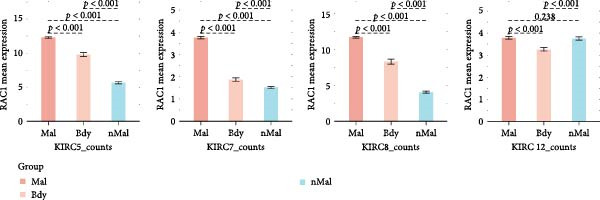
(E)

**Figure:**
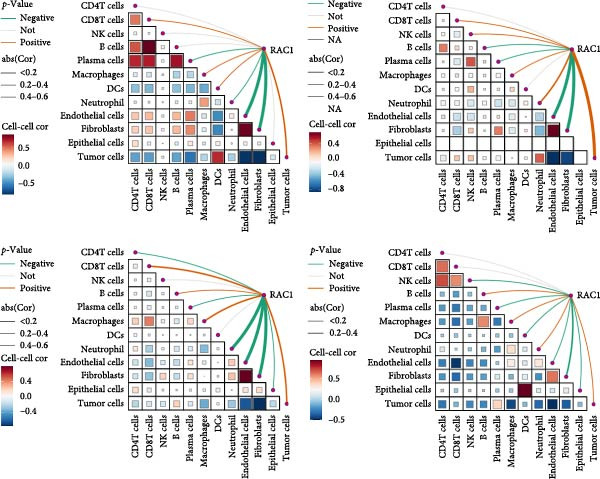
(F)

### 3.7. Integrative Immunogenomic and Oncogenic Pathway Profiling of RAC1 in ccRCC

RAC1 expression showed broad positive correlations with multiple immune checkpoint genes across functional categories, including costimulatory and coinhibitory molecules, ligands, receptors, cell adhesion molecules, and antigen presentation components, accompanied by concordant alterations in promoter methylation and copy number profiles (Figure 7A). Stratification by RAC1 expression quartiles revealed that higher RAC1 levels were associated with distinct immunogenomic landscapes, including elevated lymphocyte infiltration scores, increased TCR and BCR diversity, greater tumor mutation and neoantigen loads, higher somatic copy‐number alteration burden, and increased intratumor heterogeneity, alongside reduced tumor purity (Figure 7B). RAC1 expression was positively correlated with the activity of multiple oncogenic programs, notably EMT, proliferation, angiogenesis, inflammation, and stemness, as well as DNA damage and repair pathways (Figure 7C).G IHC analysis from the Human Protein Atlas confirmed heterogeneous protein expression of RAC1 in ccRCC, with representative cases demonstrating low or medium staining intensities (Figure 7D). Together, these results suggest that RAC1 upregulation in ccRCC is linked to an activated immune milieu, increased genomic instability, and the engagement of multiple pro‐tumorigenic signaling pathways.

Figure 7Multidimensional characterization of RAC1‐linked immune and oncogenic features in ccRCC. (A) Associations between RAC1 and immune checkpoint genes across expression, methylation, and copy number profiles. (B) Immune and genomic features across RAC1 expression quartiles. (C) Correlations between RAC1 expression and oncogenic pathway activities. (D) Representative IHC images showing low and medium RAC1 protein staining in ccRCC.

**Figure:**
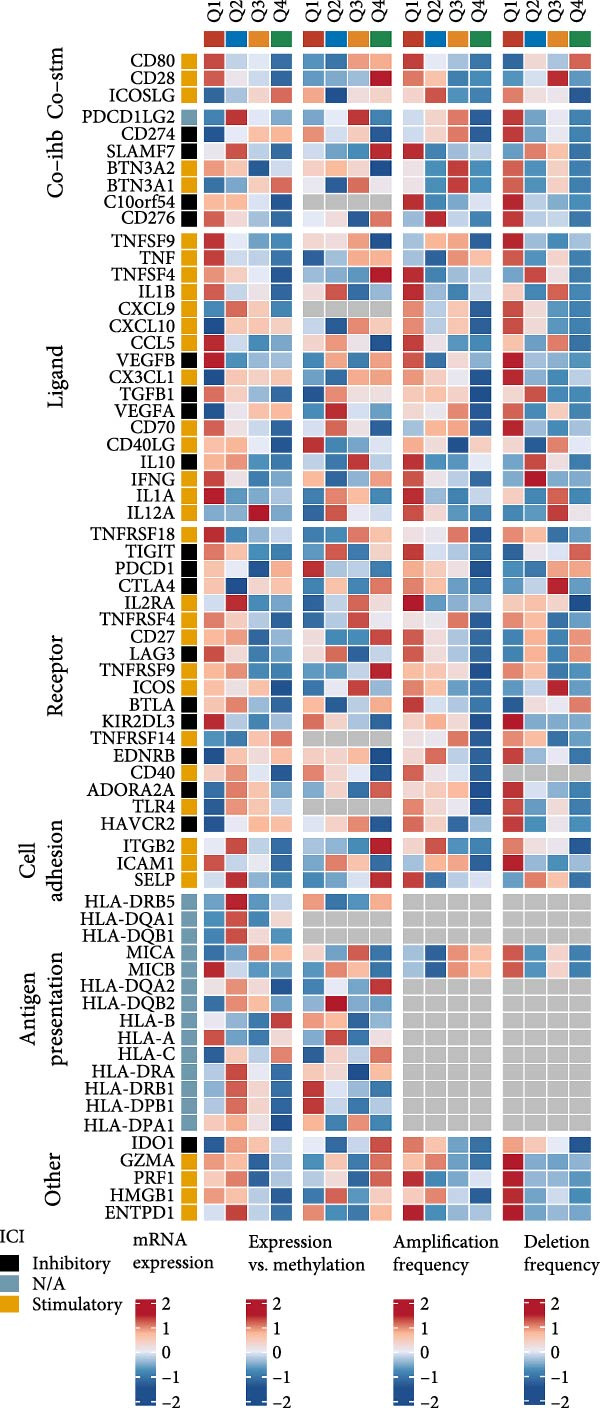
(A)

**Figure:**
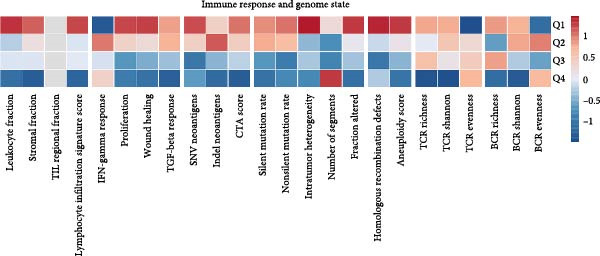
(B)

**Figure:**
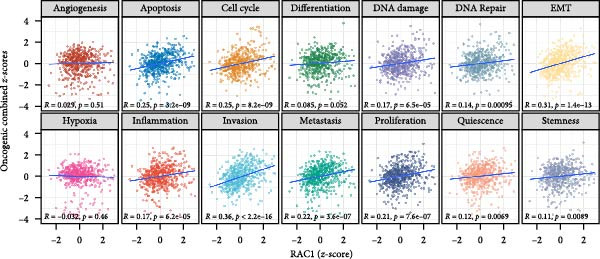
(C)

**Figure:**
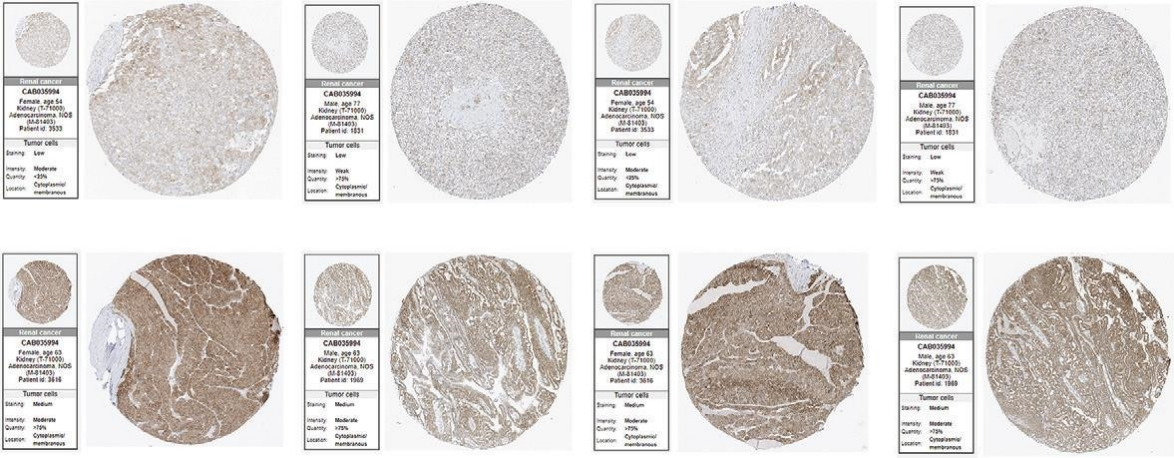
(D)

## 4. Discussion

In this study, we performed an integrated genomic and functional characterization of efferocytosis in ccRCC, demonstrating its significant prognostic value and revealing potential underlying molecular mechanisms. Efferocytosis, which involves the clearance of apoptotic cells, has been increasingly recognized for its dual role in maintaining homeostasis and promoting tumor progression through immune modulation and microenvironment remodeling [20–22]. Our findings provide evidence that enhanced efferocytosis pathway activity may be a hallmark of aggressive ccRCC, associated with advanced disease stage and poor patient survival.

One major contribution of our work is the development of a reliable prognostic model based on efferocytosis‐related genes. Utilizing a multialgorithmic approach, we identified ridge regression as the optimal method, achieving consistently superior predictive performance across multiple independent datasets. The resultant model integrates both adverse prognostic genes (e.g., RAC1, AXL, and XKR8) and protective factors (e.g., ITGAV and RHOBTB1), highlighting the complex regulatory dynamics within the efferocytosis process. Notably, this model remained robust when externally validated, underscoring its translational potential as a prognostic biomarker for ccRCC patients.

It is worth noting that the oncogenic role of RAC1 has been well documented across multiple tumor types, where it functions as a molecular switch integrating signals that regulate cytoskeletal dynamics, migration, invasion, and cross‐talk with the immune microenvironment [23]. In RCC, studies show that RAC1 expression is significantly elevated compared to adjacent normal kidney tissue and correlates with higher tumor infiltration and worse prognosis [24]. Beyond RCC, RAC1 functions as a pan‐cancer biomarker, whose overexpression is consistently associated with poor prognosis, heightened metastasis, and immunosuppressive traits, highlighting its potential as a therapeutic target [25]. Collectively, these lines of evidence provide a strong mechanistic and translational rationale for positioning RAC1 at the center of our efferocytosis‐based prognostic model in ccRCC.

Given the pivotal role of efferocytosis‐related genes in shaping tumor biology, we next focused on the key molecular determinants within our prognostic model. Among them, RAC1 emerged as the highest‐weighted gene, serving as a potential central effector linking efferocytosis to ccRCC progression, immune evasion, and oncogenic signaling pathways. Genomic analyses demonstrated that RAC1 overexpression in ccRCC is largely driven by copy number amplification, reflecting increased genomic instability in tumors with high efferocytosis activity. Such genomic alterations, including heightened TMB and distinct mutation profiles (e.g., BAP1, MTOR, TRMT1L), underscore the genetic vulnerability associated with elevated efferocytosis and highlight RAC1 as a potential genomic biomarker.

Single‐cell RNA sequencing provided deeper resolution into RAC1’s functional role, revealing its preferential enrichment in malignant cell populations. Furthermore, cell–cell communication analyses suggested that RAC1‐positive malignant cells engage in enhanced pro‐tumorigenic signaling compared to their RAC1‐negative counterparts, emphasizing its involvement in microenvironmental interactions. Spatial transcriptomic analyses corroborated these findings, demonstrating that RAC1 expression is spatially confined to tumor core regions, thereby implicating RAC1 as a central player in shaping the tumor microenvironment and possibly influencing treatment responses through localized immunosuppression or immune modulation.

Immunogenomic profiling further illuminated the broad impact of RAC1 upregulation on immune checkpoint molecules, tumor infiltrating lymphocytes, and neoantigen loads, suggesting a complex interplay between RAC1‐driven oncogenic signaling and immune surveillance mechanisms. Our integrative analyses also uncovered associations between RAC1 and multiple hallmark oncogenic pathways, such as EMT, proliferation, angiogenesis, inflammation, and DNA damage responses, aligning with previously reported roles of RAC1 in other malignancies [25, 26]. These observations substantiate the rationale for exploring therapeutic targeting of RAC1 as a novel intervention strategy for ccRCC, potentially in combination with immune checkpoint inhibitors.

Although this study integrates multiomics and single‐cell data to comprehensively characterize efferocytosis‐related mechanisms in ccRCC, several limitations should be acknowledged. The analyses were mainly based on retrospective public datasets, which may introduce batch effects and incomplete clinical annotation, necessitating validation in larger, prospective cohorts. The efferocytosis‐related pathways were derived from existing gene sets and may not capture all relevant or noncanonical components. The single‐cell and spatial datasets analyzed are also limited in sample size and resolution, and future integration of higher‐resolution and spatially comprehensive data will be valuable for refining these findings. Moreover, the associations between RAC1 alterations, efferocytosis activity, and tumor progression remain correlative and require further mechanistic experiments to establish causality and clarify the underlying biological pathways.

In conclusion, our comprehensive characterization establishes efferocytosis, particularly through RAC1‐driven mechanisms, as a crucial contributor to ccRCC aggressiveness and poor prognosis. By integrating genomic, transcriptomic, spatial, and immunological dimensions, this study not only provides novel insights into ccRCC pathogenesis but also suggests promising diagnostic biomarkers and therapeutic targets. Further clinical and experimental validations are essential to translate these insights into improved patient outcomes.

## Ethics Statement

The authors have nothing to report.

## Consent

The authors have nothing to report.

## Disclosure

Shuijie Shen and Mingcong Zhang approved the final manuscript.

## Conflicts of Interest

The authors declare no conflicts of interest.

## Author Contributions

Bing Shi, Minghao Deng, Jiakang Ma, and Chao Chen contributed equally to this work. Bing Shi and Minghao Deng designed the study, performed data analysis, and wrote the manuscript. Jiakang Ma and Chao Chen provided critical revisions and supervision. Aijin Peng, Anli Zhu, and Rongchao Yang contributed to data analysis and manuscript drafting. Zhenhua Jin provided statistical support. Jian Zhu contributed to study design. Shuijie Shen and Mingcong Zhang supervised the project.

## Funding

This work was supported by the Key R&D and Promotion Projects of Henan Province (Grant 232102310130) and the Medical and Health Research Project of Zhejiang Province (Grant 2022RC250).

## Supporting Information

Additional supporting information can be found online in the Supporting Information section.

## Supporting information


**Supporting Information 1** Table S1: List of 192 efferocytosis‐related genes curated from the Molecular Signatures Database.

**Supporting Information 2** Table S2: Ridge regression coefficients of the efferocytosis‐related prognostic model.

## Data Availability

All data supporting the findings of this study are available within the article.
